# *In vitro *modulation of inflammatory cytokine and IgG levels by extracts of *Perna canaliculus*

**DOI:** 10.1186/1472-6882-6-1

**Published:** 2006-01-13

**Authors:** Sachin Mani, John W Lawson

**Affiliations:** 1 Department of Microbiology and Molecular Medicine, Clemson University Clemson, SC 29634, USA

## Abstract

**Background:**

Inflammation is a predominant characteristic of autoimmune diseases which is characterized by the increased expression of pro-inflammatory cytokines. Soon to be published work from our laboratory has shown that ingestion of *Perna canaliculus *prevents the development of autoimmune diseases such as Systemic Lupus Erythematosus and rheumatoid arthritis in laboratory animals. The current paper attempts to illustrate how Perna can alleviate inflammation by modulating inflammatory cytokines, cyclooxygenase enzymes and Immunoglobulin-G (IgG) levels.

**Methods:**

In the present study, hydrochloric acid [HCl] and Tween-20 were used to develop extracts of Perna. These extracts were assayed for protein content. Increasing concentrations of these extracts were then tested in cell culture for modulation of inflammatory cytokine, cyclooxygenase enzymes and IgG levels. Parallel tests were run using an available glycogen extract of Perna as a comparison to our in-house laboratory preparations.

**Results:**

Tween-20 Perna extracts were found to be more stable and less toxic in cell culture than HCl digest of Perna. They also assayed higher in protein content that HCl extracts. Although both extracts inhibited IgG production in V2E9 hybridomas, Tween-20 extracts were more consistent in IgG suppression than HCl extracts. Overall Tween-20 extracts effectively decreased levels of TNF-α, IL-1, IL-2 and IL-6 as observed using cytokine bioassays. Twenty micrograms of Tween-20 Perna extracts induced such significant decreases in inflammatory cytokine production that when tested on sensitive cell lines, they very nearly abolished the decrease in viability induced by these cytokines. Tween-20 extracts effectively inhibited both COX-1 and COX-2 cyclooxygenase activity. As a comparison, the glycogen extract also demonstrated a similar though weaker effect on COX-1 and COX-2 enzymes. The active components of both extracts (Tween-20 and glycogen) were observed to possess molecular weights above 100 kDa. Although the anti-cytokine activity of the Tween-20 extract was destroyed by Proteinase-K treatment, the anti-COX-1 and anti-COX-2 activity of both the extracts were not sensitive to protease treatment.

**Conclusion:**

We have successfully demonstrated modulation in the levels of inflammatory cytokines, cyclooxygenase enzymes and immunoglobulins by our in-house laboratory preparations of *Perna canaliculus*, whereby suggesting an immunomodulatory role of *Perna canaliculus *in regulating inflammation.

## Background

Autoimmune diseases include a wide spectrum of systemic diseases such as systemic lupus erythematosus (SLE) and rheumatoid arthritis (RA) [1,2]. The important characteristic of all autoimmune syndromes is inflammation and the damage inflicted upon self-tissues mediated by host cellular and/or humoral responses. Autoantibodies are fundamental to all autoimmune diseases and are products of auto-reactive B cells and plasma cells, which in turn, are driven by a T helper subset 1 (Th1) immune response [3]. T lymphocytes play a role in the disease process by the stimulation of other immune cells via T helper subset 2 (Th2) and/or direct cell-cell interaction [4-6]. Cytokines also play a role in autoimmunity by stimulating or regulating B and T cells, macrophages, and/or dendritic cellular responses to antigenic stimulation [7]. It is well established that inflammatory cytokines play important roles in the development and pathogenesis of autoimmune diseases such as RA and SLE [8-11]. These factors overcharge or overwork the immune networks and must be regulated by modulation or suppression. The New Zealand green-lipped mussel (*Perna canaliculus*) contains components that can act as natural immunomodulators. Laboratory investigations of the freeze-dried extract of *Perna canaliculus *have demonstrated that Perna extracts decreased experimentally induced inflammation in carrageenan-induced edema of the rat hind footpad [12]. In our laboratory, Perna has been shown to suppress the development of collagen-induced arthritis (CIA) in the rat, and has even reversed inflammation associated with CIA in mice. Perna has been employed as a therapeutic agent in arthritis with some degree of success [13-15]. However, evidence from other clinical trials utilizing Perna products for rheumatoid arthritis (RA) and osteoarthritis (OA) have been conflicting with respect to the therapeutic potential of Perna [14,16].

The exact mechanism of Perna's anti-inflammatory action is unknown. There is some evidence that certain components in Perna may inhibit prostaglandin synthesis via production of prostaglandin synthetase inhibitors [17-19]. Perna also contain the histamine blocker lysolecithin that may contribute to its anti-inflammatory activity [20]. Omega 3 fatty acids in *Perna canaliculus *have been shown to decrease inflammation [21,22]. Although many authors have demonstrated various anti-inflammatory mediators in *Perna canaliculus*, inconsistencies in therapeutic activity levels persist. These inconsistencies may be due to the lack of standardized extraction / fractionation protocols and the possible presence of many anti-inflammatory mediators in *Perna canaliculus*. Different approaches to extract and/or fractionate Perna have been employed albeit with mild success [20-24].

Our paper discusses two extraction techniques for obtaining Perna extracts and explores the effects of these extracts on inflammatory cytokine production, cyclooxygenase activity and IgG modulation in both lymphocytic cell lines and B cell hybridomas.

## Methods

### Preparation of *Perna canaliculus *(Perna) extracts

**Figure 1:**
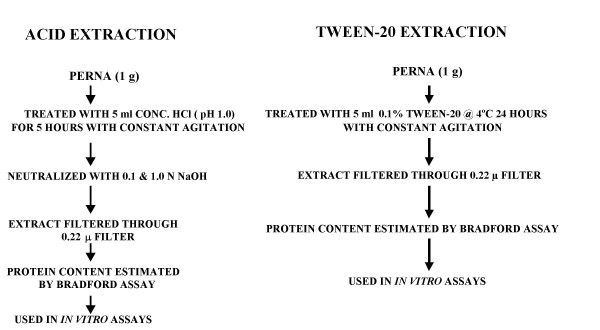
Protocols for preparing extracts of Perna powder, a freeze dried preparation of Perna canaliculus using either concentrated HCl or 0.1% Tween-20.

We used two protocols to create extracts of the lyophilized Perna powder (FoodScience Corp., Essex Jct., VT), the commercially available freeze-dried powder of the New Zealand green lipped mussel, *Perna canaliculus*. The first method used concentrated hydrochloric acid (1 M HCl) while the second employed Tween-20 (Figure 1). Acid extraction proceeded by placing 1 g samples of freeze-dried Perna powder in 250 ml beakers along with 5 ml of concentrated HCl solution (pH 1.0) in a 37°C water bath for 5 hours with constant agitation. The extracts were neutralized with either 0.1 N or 1.0 N sodium hydroxide (NaOH) and filtered through a series of filters leading to 0.22μ. The filtrate was then analyzed and adjusted for protein content by the Bradford assay prior to use in cell culture. Tween-20 extraction of Perna was performed by treating 1 g samples of freeze-dried Perna with 5 ml of 0.1% Tween-20 solution for 24 hours at 4°C with constant agitation. The samples were then filtered and assayed for protein content as above before use in cell culture. Both HCl and Tween-20 extracts were adjusted to yield a range of concentrations for use in cell culture assays based on their protein content.

### Cell Lines and Cell Culture

The cell lines used in this study were V2E9, THP-1, L-929, U-937, A375.S2, Jurkat E6-1, EL-4, CTLL-2, LS174T, and 7TD1. Characteristics of each cell line including cytokine production and/or sensitivity are presented in Table 1. All the cell lines, with the exception of V2E9, were obtained from the American Type Culture Collection (ATCC). V2E9 cell line, a B cell hybridoma constitutively secreting mouse IgG against the β1 subunit of chicken integrin, was obtained courtesy of Ellis Kline, Clemson University. V2E9 cells grown in Ex-Cell 610 medium (JRH Biosciences) supplemented in initial studies with 10% fetal bovine serum (FBS, Gibco BRL) and 1% Antibiotic-Antimycotic (Gibco BRL) and later adapted to grow in serum-free media. All the other cell lines were cultured as per ATCC recommendations with regard to media and supplements. CTLL-2 and 7TD1 are cytokine-dependent cell lines requiring the addition of Interleukin-2 (IL-2) and IL-6 respectively. All cell lines were grown at 37°C in a humidified 5% CO_2 _incubator, except for EL-4 mouse thymoma cells which required 10% CO_2_.

**Table 1:** List of cytokine producing cell lines and their corresponding responder cell lines

**CELL LINES**	**CYTOKINES PRODUCED**	**RESPONDER CELL LINES**
THP-1	TNF-α	L-929 (sensitive to TNF-α)
U-937	IL-1	A375.S2 (sensitive to IL-1)
Jurkat E6-1	IL-2	CTLL-2 (Proliferates in presence of IL-2)
		HT-2 (Proliferates in presence of IL-2)
EL-4	IL-2	CTLL-2 (Proliferates in presence of IL-2)
		HT-2 (Proliferates in presence of IL-2)
LS 174T	IL-6	7TD1 (Proliferates in presence of IL-6)

### Modulation of IgG production in V2E9 hybridoma cells (ELISA)

V2E9 cells were harvested, enumerated, and 1 ml of cells in Ex-cell 610 media at a concentration of 5e^6 ^cells/ml were transferred into 24 well plates. One hundred μl of varying dosages (containing 0 μg, 5 μg, 10 μg, 15 μg, 20 μg, and 25 μg protein) of Perna extract were added. An additional 900 μl of Ex-cell media were added to bring the total volume to 2 ml. After incubation at 37°C for 48 hours, supernatants of Perna-treated and untreated control cells were drawn off and assayed for IgG levels using ELISA.

Goat affinity purified antibody to mouse IgG (Cappel, Organon Teknika Corp.) was diluted in phosphate buffered saline [PBS] (pH 7.4) to a concentration of 0.1 μg/μl. Ninety-six well, high-affinity binding ELISA plates (Corning, NY) were coated with 100 μl of this buffered suspension for an hour at 37°C. The plates were placed for an additional hour at room temperature (RT). The plates were washed twice with PBS, blocked with freshly prepared Blotto (Skim milk in PBS) for 60 minutes at 37°C, rinsed twice with PBS, and blotted. One hundred μl of the supernatants from the treatment and control wells were added to the plates and allowed to incubate for an hour at 37°C. Following incubation, plates were rinsed twice with Blotto and PBS. One hundred μl of peroxidase conjugate goat affinity-purified antibody to mouse IgG (Cappel, Organon Teknika Corp.) diluted 1:100 in PBS were added to each well. Plates were incubated for 30 minutes at 37°C and rinsed four times with Blotto and twice with PBS. One hundred micro liters of fresh color developer [24.3 ml of 0.1 M citric acid (Sigma), 25.7 ml of 0.2 M Na_2_HPO_4 _(Fisher Scientific), 50 ml distilled water, 40 mg o-phenylenediamine (Sigma) and 40 μl of 30% H_2_O_2 _(Fisher Scientific)] was added to each well and allowed to react for 20 minutes in the dark at RT. Reactions were terminated by the addition of 50 μl of 2.5 M H_2_SO_4 _to each well. The plates were read using a BIO-RAD benchmark microplate reader at 490 nm.

### Modulation of cytokine production in cell lines (Cytokine Bioassays)

Modulation of inflammatory cytokine production by Perna was determined by cytokine bioassays [25-28]. Supernatants of Perna-treated and untreated cytokine producing cell lines (producers) were added to indicator cell lines specifically sensitive to one of the cytokines. Proliferation or inhibition of these indicator cell lines signals the extent of cytokine secretion by the producer cell lines. The optimum cell number per well for each cell line was determined by titration. The producer cell lines which grow in cell suspension, THP-1, Jurkat E6-1, EL-4 and U-937, were plated on 48 well plates at a concentration of 5e^6 ^cells/well, whereas the adherent producer cell line, LS174T, was plated at a concentration of 5e^5 ^cells/well. Suspension responder cell lines (CTLL-2 and 7TD-1) were added at a concentration of 1e^6 ^cells/well in a 96 well plate, whereas the adherent responder cell lines, L-929 and A375.S2, were plated at concentrations of 3e^4 ^cells/well and 4e^4 ^cells/well respectively. THP-1 cells, which secrete tumor necrosis factor-alpha (TNF-α) when stimulated with lipopolysaccharide (LPS), were treated with varying concentrations (containing 0 μg, 5 μg, 10 μg, 15 μg, 20 μg, and 25 μg protein) of Perna for a period of 24 hours at 37°C in a humidified 5% CO_2 _incubator. Supernatants from these cells were then added to the indicator cell line L929 that is sensitive to TNF-α and incubated for 24 hours at 37°C in a humidified 5% CO_2 _incubator [29-31]. After incubation, MTS-PMS solution, (Cell Titer 96 Aqueous Kit, Promega, Madison, WI) was added to each well as recommended by the manufacturer [32]. Detection of cell proliferation or inhibition is based on a colorimetric assay system utilizing the tetrazolium reagent, MTS. MTS is reduced to a water soluble formazan dye, via the alternate electron acceptor PMS (phenazine methosulphate) in the mitochondria of living cells. Samples are read at 4 hours using a BIO-RAD benchmark microplate reader at 490 nm. The amount of color produced is directly proportional to the number of viable cells. The experiments were carried out in triplicate and repeated three to six times for each cytokine. Similarly, U-937 cells which secrete Interleukin-1 (IL-1) constitutively were treated with Perna and the supernatants added to the sensitive cell line A375.S2 [33,34]. Likewise, Jurkat E6-1 and EL-4 mouse thymoma cells which when stimulated with Ionomycin and phorbol myristate acetate (PMA) respectively secrete IL-2, were treated with Perna and the supernatants were added to the responder cell line CTLL-2 [35-38]. Finally, LS174T colon adenocarcinoma cells that constitutively secrete IL-6 were treated with the Perna extracts and the supernatants added to the responder 7TD1 cell line [39-41].

### Cyclooxygenase-1 (COX-1) and Cyclooxygenase-2 (COX-2) Assay

COX-1 and COX-2 assays were performed using the colorimetric Ovine Cyclooxygenase (COX) assay kit (Cayman Chemical Company). Different dilutions of Perna extracts (Tween-20 & Glycogen) were aliquoted into microfuge tubes and lyophilized. The lyophilized samples were then resuspended in Dimethyl Sulfoxide (DMSO) and diluted further to derive the appropriate concentrations for testing. The assay was then performed as described in the assay kit booklet.

### Statistical analysis

The results were expressed as the means ± SEM of the number of measurements (independent experiments) shown. Statistical comparisons between dosage groups were done using the paired t-test and a one-way ANOVA, with *P *< 0.01 indicating significance.

## Results

### *Perna canaliculus *(Perna) extracts

Extracts of Perna was prepared using two procedures, one, which uses concentrated HCl and the other a cationic detergent, Tween-20 (Figure 1). Hydrochloric acid was chosen initially for preparing an extract of Perna to simulate the acidic gastric conditions of Perna when it is taken orally. Tween-20 which draws out membrane-bound proteins was used on the premise that the active component in Perna might be a protein moiety [22]. It was found that Tween-20 extract yielded higher protein recovery levels than HCl extract. Protein estimation was used as a quantitative measure of extract recovery at the end of HCl digestion and Tween-20 treatment and also as a standard for measuring activity. The low amount of protein in HCl extracts could be based on either the dilution effect of neutralizing HCl with NaOH or the inability of HCl to extract active proteins efficiently. Since protein content was used as the standard to measure activity, HCl extracts had to be concentrated prior to use in cell culture assays. Extracts in the range of 0 μg, 5 μg, 10 μg, 15 μg, 20 μg, and 25 μg protein content were used in assays to determine activity. The neutralized HCl extracts were toxic to the cells at concentrations approaching 20 μg (~70–80% viability), whereas Tween-20 extracts did not affect viability of cells (>90% viability) even at concentrations greater than 25 μg.

As a comparison to our in-house laboratory prepared extracts, a commercially available extract of Perna canaliculus was obtained from Aroma New Zealand Limited, Christchurch, New Zealand. This extract is rich in glycogen and obtained by enzymatic treatment and extraction with aqueous ethanol. Insoluble components were separated by centrifugation and the extract isolated from the supernatant by lyophilization [42].

### Modulation of IgG production in V2E9 hybridoma cells

**Figure 2:**
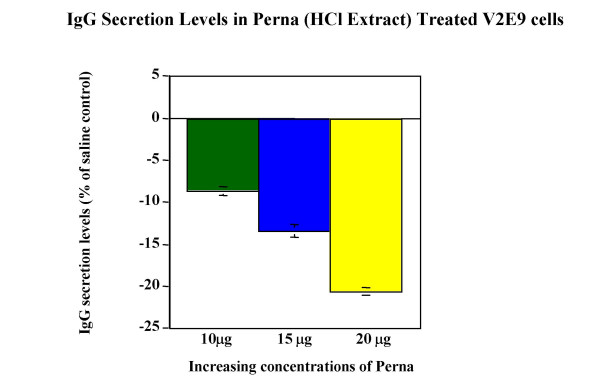
Dose response effects of Perna (HCl extract) on IgG secretion in V2E9 hybridoma cells. Each bars represents the mean ± SEM of data points from three independent experiments with triplicate wells. Columns representing 10 μg, 15 μg and 20 μg Perna concentrations are statistically different from the saline control (no Perna). P ≤ 0.01.

**Figure 3:**
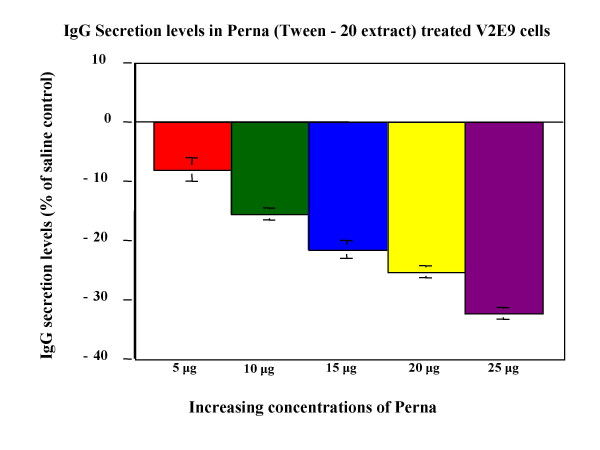
Dose response effects of Perna (Tween-20 extract) on IgG secretion levels in V2E9 hybridoma cells. Each bar represents the mean ± SEM of data points from three independent experiments with triplicate wells. Columns representing 5 μg, 10 μg, 15 μg, 20 μg and 25 μg Perna concentrations are statistically different from the saline control (no Perna). P ≤ 0.01.

**Figure 4:**
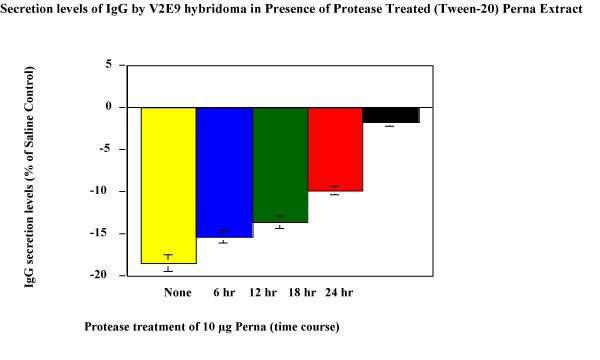
Effect of time course dependent protease treated Perna (Tween-20 extract) on IgG secretion levels in V2E9 hybridoma cells. Each bar represents the mean ± SEM of data points from three independent experiments with triplicate wells. Columns representing 6 hr, 18 hr and 24 hr protease treatments are statistically different from the saline control (no Perna). P ≤ 0.01.

Both HCl and Tween-20 extracts were compared for the ability to modulate IgG antibody production in V2E9 hybridoma. Both extracts significantly decreased antibody expression in the hybridoma (Figure 2, Figure 3). Maximum decreases of antibody levels (21%) were observed for HCl extract containing 20 μg of protein, whereas Tween-20 extract decreased antibody levels by 26% at similar protein concentrations. To determine whether the active component of Perna was associated with a protein moiety, protease digestion with Proteinase-K of Tween-20 extracts from Perna was carried out over different time periods. On addition of these digests to V2E9 cells, no decrease in antibody production was observed (Figure 4). Furthermore the decrease in inhibitory activity of Proteinase-K treated Perna extract was time dependent. IgG secretion levels increased in parallel with the length of protease pretreatment. This correlated with an increase in IgG levels over baseline levels from -18.5% to -1.6%, over a 48-hour protease treatment period. The proteolytic enzyme apparently eliminated the component in the Perna extract, which inhibited antibody production.

### Modulation of inflammatory cytokine expression

**Figure 5:**
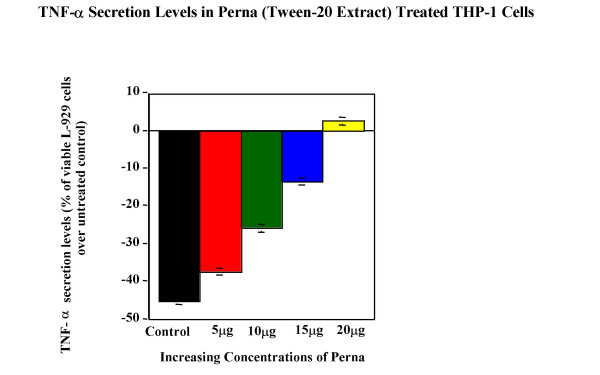
Dose response effects of Perna (Tween-20 extract) on TNF-α secretion levels in THP-1 cell line. Each bar represents the mean ± SEM of data points from three independent experiments with triplicate wells. Columns representing the different concentrations are all statistically different from the stimulated but untreated control (no Perna). P ≤ 0.01.

**Figure 6:**
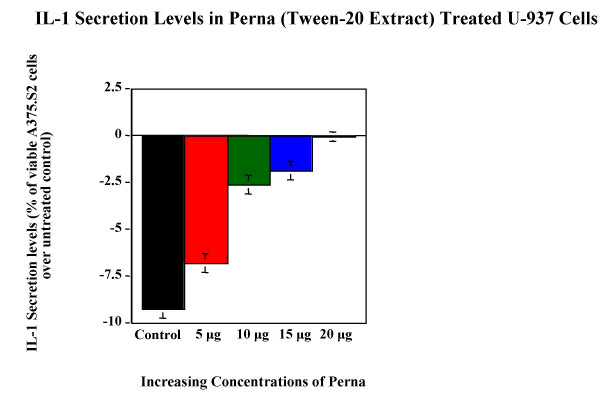
Dose response effects of Perna (Tween-20 extract) on IL-1 secretion levels in U-937 cell line. Each bar represents the mean ± SEM of data points from three independent experiments with triplicate wells. Columns representing the different concentrations are statistically different from the stimulated but untreated baseline controls (no Perna). P ≤ 0.01.

**Figure 7:**
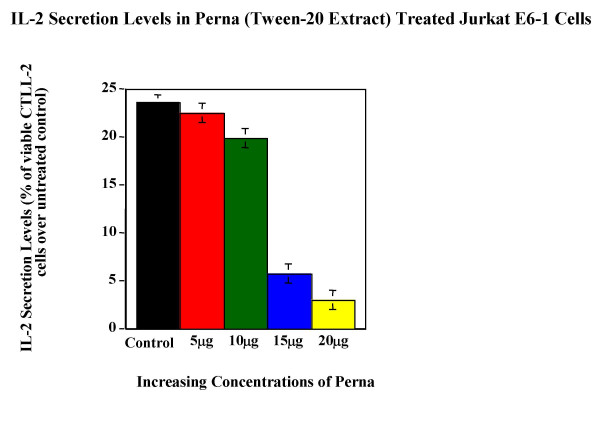
Dose response effects of Perna (Tween-20 extract) on IL-2 secretion levels in Jurkat E6-1 cell line. Each bar represents the mean ± SEM of data points from three independent experiments with triplicate wells. Columns representing 10 μg, 15 μg and 20- μg concentrations of Perna are statistically different from the stimuated but untreated baseline control (no Perna). P ≤ 0.01.

**Figure 8:**
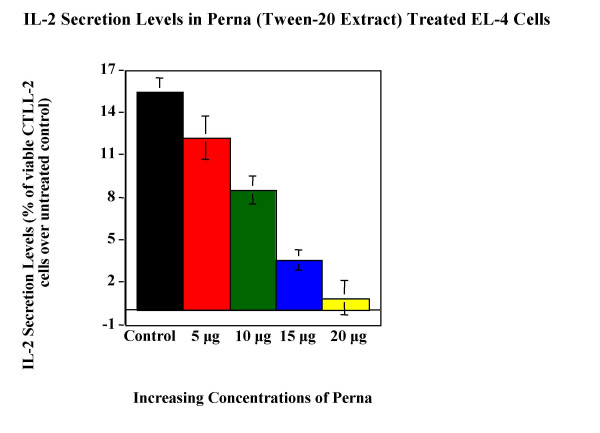
Dose response effects of Perna (Tween-20 extract) on IL-2 secretion levels in EL-4 mouse thymoma cells. Each bar represents the mean ± SEM of data points from three independent experiments with triplicate wells. Columns representing the different concentrations are statistically different from the stimuated but untreated baseline control (no Perna). P ≤ 0.01.

**Figure 9:**
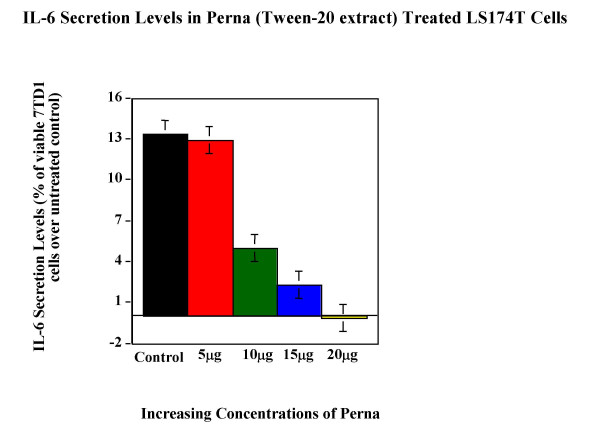
Dose response effects of Perna (Tween-20 extract) on IL-6 secretion levels in LS 174T cell line. Each bar represents the mean ± SEM of data points from three independent experiments with triplicate wells. Columns representing 10 μg, 15 μg and 20- μg concentrations of Perna are statistically different from the stimuated but untreated baseline control (no Perna). P ≤ 0.01.

Tween-20 extract of Perna was tested for its ability to modulate cytokine expression in cell lines. Cytokine bioassay results (Figure 5, Figure 6, Figure 7, Figure 8 & Figure 9) demonstrated the dose dependent effect of the Perna extract in decreasing inflammatory cytokine levels. The maximal effect for TNF-α was observed with 20 μg of Perna extract wherein the cytokine level was decreased to 48% of control levels as observed by the proliferation rate of L-929 cells (Figure 5). Similarly, in U-937 cells, which secrete IL-1, as the concentration of Perna extract expressed as protein increased from 5 μg to 20 μg, the level of IL-1 secretion decreased as detected by an increase in the viability and proliferation of A375.S2 cells (Figure 6). This corresponds to approximately a 10% decrease in IL-1 levels over baseline controls following treatment with 20 μg of Perna. Comparable results were obtained with Perna-treated Jurkat E6-1 and EL-4 cells, which secrete IL-2. Supernatants from these cell lines, when added to the responder cell line CTLL-2, triggered a decline in proliferation of CTLL-2 due to decreased levels of IL-2. In Jurkat E6-1, secretion levels of IL-2 as evidenced by the proliferation of CTLL-2 cells dropped from a high of 23.56% to a low of 2.98% (Figure 7). This translates to a drop of 21% with 20μg of Perna extract. In EL-4 mouse thymoma cells, IL-2 secretion levels as measured by the growth rate of CTLL-2 cells reduced from 15.38% to 0.89% with the highest growth rate drop of 14% observed with 20 μg Perna (Figure 8). Likewise IL-6 production levels in LS174T were also depressed in the presence of Perna extract, leading to a drop in proliferation of 13% in 7TD1 cells (Figure 9).

### Cyclooxygenase-1 (COX-1) and Cyclooxygenase-2 (COX-2) Assay

**Figure 10:**
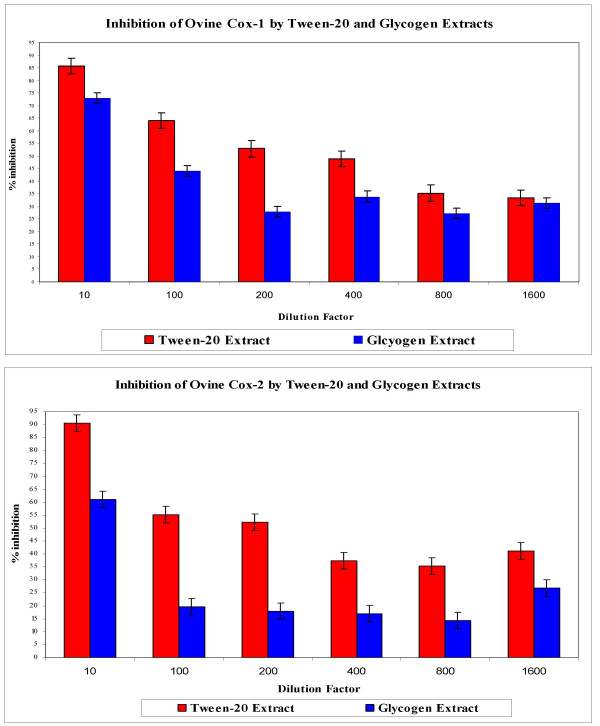
50% inhibition of Cox-1 (Figure A.) and Cox-2 (Figure B.) was seen between the 1:200 and 1:400 dilutions of the Tween-20 extract and between the 1:10 and 1:100 dilutions of the Glycogen Extract.

We examined the effects of the Tween-20 extract of Perna on the activity of both COX-1 and COX-2 enzymes. Included in parallel studies was the glycogen rich commercial extract derived from Aroma New Zealand. As observed in figure 10, COX-1 activity was inhibited by over 50%, when Perna extracts were tested. The magnitude of COX-1 inhibition was higher in dilutions from Tween-20 extracts (1:200 and 1:400 dilutions) compared to the Glycogen extracts (1:10 and 1:100 dilutions). Inhibition of both the COX-1 and COX-2 enzyme activity was consistently higher in all Tween-20 preparations (Figure 10).

## Discussion

Inflammation is a prominent feature of autoimmunity leading to tissue injury. Cytokines play an important role in inflammatory autoimmune diseases such as RA and SLE [43]. Elevated local and systemic levels of inflammatory cytokines are often associated with depressed levels of anti-inflammatory cytokines, which suppress inflammatory pathways suggesting an aberrant Th1/Th2 regulation. Current research on immunomodulation focuses on the ability of agents to regulate or modify the immune network via regulation of Th1/Th2 immune responses. Natural substances such as hormones, vitamins, and amino acids have been used to achieve this modulation [44,45].

*Perna canaliculus *has been a popular nutritional supplement for treating arthritis. Oral consumption of Perna stems from the observation that native New Zealanders (Maoris) who lived near the sea had a much lower incidence of arthritis than their relatives who lived inland [46]. Both the diets were similar with the exception that the Maoris on the coast consumed large amounts of green-lipped mussel. Various researchers have experimented with a range of methodologies for generating *Perna canaliculus *extracts and have demonstrated the presence of various anti-inflammatory mediators [21,22,47,48]. Inconsistencies in the therapeutic availability of the active components in these preparations still persist and may be due to the presence of many anti-inflammatory mediators in *Perna canaliculus*. Different approaches to extract and/or fractionate Perna have been employed albeit with mild success [20-24]. Hence, we developed simple extraction protocols using HCl and Tween-20 for generating soluble extracts of the commercially available freeze-dried powder of *Perna canaliculus *as a means to isolate, test and identify some of the active components. Our rationale behind using Hydrochloric acid (HCl) as an extraction tool stems from our understanding of the primary route of Perna intake which is ingestion. This is based primarily on our laboratory studies related to collagen induced arthritis (CIA) experiments involving rats and mice, where we observed a delay in the onset of arthritis in animals that were given Perna orally (manuscript in preparation). Similar observations have been made by other investigators with the oral administration of either *Perna canaliculus *or Perna extracts in alleviating symptoms of arthritis and other autoimmune diseases [47-51] These observations led to the assumption that the active principle may be facilitated by the digestive process. Hence we simulated the acidic environment of the stomach by using hydrochloric acid (HCl) to extract Perna. Tween-20 is a cationic detergent and was used based on the assumption and prior evidence from Miller *et al*., that the anti-inflammatory principle may be associated with a protein moiety [22]. We found that Tween-20 extraction of Perna was more stable and less toxic in cell culture than HCl digest of Perna. The total protein content of HCl extract was lower and more variable than Tween-20 extract due to the elaborate nature of the HCl extraction process. Neutralization with NaOH after HCl treatment dilutes this extract, requiring removal of water by lyophilization to achieve parity in protein content prior to use in cellular assays. This concentration process led to an increase in osmolarity and contributed towards decreased cellular viability. Tween-20 being a cationic detergent and when used at a concentration of 0.1% did not affect the viability of cells (>90% viability) even at standard protein concentrations as high as 25 μg. Thus, Tween-20 extracts was the preferred method of extraction in all cytokine bioassays. Although the extract derived from HCl digestion of Perna was not as active as that obtained from similar treatment with Tween-20, our results demonstrate that the anti- inflammatory properties were conserved in both extraction processes.

Perna extracts inhibited IgG production in the V2E9 hybridoma cell line. Tween-20 extract was more consistent in IgG suppression than the HCl fraction. In RA and SLE, the pathological antibody associated with disease manifestations is of the IgG2a isotype which is produced under the influences of TNF-α and IL-1, mediators of the inflammatory T-helper 1 (Th1) cellular response. Although there is no direct correlation between IgG levels of V2E9 hybridoma and pathological antibody of autoimmune disease, we infer that decreases in IgG levels may mirror an improvement in pathological antibody.

Cytokine bioassays were used to ascertain a role for Perna in modulating inflammatory cytokine production. Overall Tween-20 extracts effectively decreased levels of TNF-α, IL-1, IL-2 and IL-6. Twenty μg of Tween-20 Perna extracts induced significant decreases in inflammatory cytokine production, which when tested on sensitive cell lines, very nearly abolished the decrease in viability induced by most inflammatory cytokines (TNF-a, IL-1 & IL-6). Although some of the changes in cytokine expression reported via the bioassay may appear to be numerically small, they represent relatively large changes in percentage. In the case of IL-1, the level of IL-1 production in U-937 producer cell line led to a 10% decrease in viability of the sensitive responder cell line (A375.S2). This 10% decrease in A375.S2 viability is reflective of the level of IL-1 produced by U-937. This is the control expression level of IL-1 and termed as IL-1 control, where there was no Perna treatment. Using this as our baseline level for normal IL-1 expression, we found that increasing concentrations of Tween-20 Perna extracts led to an increase in responder cell (A375.S2) viability. In other words, 10 μg of Tween-20 Perna extract decreased the levels of IL-1 secretion in U-937 which in turn led to a 2.5% decrease in A375.S2 viability as compared to an observed 10% decrease in the control. This translates to at least 4-fold decrease in IL-1 expression levels. Similarly when 20 μg of Perna extract was tested in the bioassay, the viability of A375.S2 increased near 100%. This lack of decrease in viability of A375.S2 is reflective of the absence of any IL-1 production by U-937 cell line in presence of 20 μg of Perna extracts. This observed decrease in IL-1 expression although small as measured by cytokine bioassays was repeatable in at least four independent experiments. Minor cytokine changes tend to become significant when complex networked systems in living organisms are involved. Similar changes in cytokine profiles that are enough to alter the therapeutic outcome have been observed in mouse models of rheumatoid arthritis and Systemic lupus erythematosus [52]. This decrease in inflammatory cytokines may help to explain the delayed onset of arthritis and the reversal of arthritic inflammation observed in the collagen induced arthritis (CIA) experiments involving rats and mice in our laboratory (manuscript in preparation). The ability of Perna to decrease inflammatory cytokine levels is relevant in control of Th1 mediated immune responses. The effect of Perna in decreasing IgG levels in V2E9 associated with observed decreases in inflammatory cytokine production might mimic amelioration of pathological factors in autoimmune diseases.

Treatment of the extract with protease removed the active component of the soluble fraction and made Perna ineffective in modulating IgG production. This suggests that the active component in Perna may be associated with a protein moiety. This corroborates with the work done by Miller *et al*., who showed Perna to possess an anti-inflammatory glycogen component associated with a protein moiety, probably as a glycoprotein [22].

We used a commercially available extract of Perna canaliculus obtained from Aroma New Zealand Limited, Christchurch, New Zealand as a comparison to our in-house laboratory preparations. This extract is reported to be rich in glycogen and has been obtained by enzymatic treatment followed by extraction with aqueous ethanol and is now called the Glycogen extract [42]. This glycogen extract was found to decrease foot pad edema (inflammatory edema) which had been induced by carrageen in rats [22]. In preliminary studies we found that the active component of both the Tween-20 and glycogen extracts had molecular weights above 100 kDa. Anti-inflammatory cytokine activity was destroyed on treatment of the Tween-20 extract with Proteinase-K. A similar digestion eliminated the anti-inflammatory activity of the glycogen extract in the Carrageen induced edema model in rats [22].

In evaluating the anti-inflammatory potential of our Perna extracts, we have performed the colorimetric Ovine Cyclooxygenase (COX) assay. We have successfully demonstrated that Tween-20 extracts of *Perna canaliculus *was effective in inhibiting both COX-1 and COX-2 cyclooxygenase activity. As a comparison, the glycogen extract also demonstrated a similar though weaker effect on COX-1 and COX-2 enzymes. COX enzymes are essential in the inflammatory process and control the downstream regulation of immune cell activation and inflammatory cytokine induction [53]. Currently a great deal of attention is being paid to understanding the multifaceted nature of the cyclooxygenase and the lipooxygenase pathways in inflammation and cytokine induction. Our results demonstrating the inhibition of Ovine COX-1 and COX-2 have supported the role played by Perna extracts in modulating inflammation and inflammatory cytokine response.

## Conclusion

In summary, we have prepared two distinct extracts of the freeze dried Perna powder. The Tween-20 extract was determined to be more potent and less toxic than HCl extract in decreasing IgG expression in hybridoma cell culture. Consequently we used the Tween-20 extracts in all subsequent experiments involving modulations of inflammatory cytokine expression and cyclooxygenase activity. Tween-20 Perna extracts were shown to suppress pro-inflammatory cytokines expression, inhibit COX-1 and COX-2 activity and decrease IgG levels. In parallel studies using glycogen extracts, we observed similar decline in COX-1 and COX-2 activity. Although, both the extracts (glycogen and Tween-20) possessed active components that appeared to be larger than 100 kDa, the component effective against inflammatory cytokines was sensitive to proteolysis using Proteinase-K and Pronase, whereas the component effective against COX enzymes was resistant to proteolysis. Based on all the above observations, we suggest a possible immunomodulatory role for *Perna canaliculus *in mediating Th1/Th2 regulation as it relates to inflammation.

## Competing interests

The author(s) declare that they have no competing interests.

## Authors' contributions

Both authors contributed equally and have read and approved the final manuscript.

## Pre-publication history

The pre-publication history for this paper can be accessed here:


